# Intra-articular corticosteroid therapy in oligoarticular juvenile arthritis: our clinical experience

**DOI:** 10.1186/1546-0096-9-S1-P200

**Published:** 2011-09-14

**Authors:** C Trigilia, T Timpanaro, G Caff, G Belfiore, V Pavone, P Barone, R Garozzo

**Affiliations:** 1 Pediatrics Department, University of Catania, Catania, Italy; 2 Orthopedics Department, University of Catania, Catania, Italy

## Background

Oligoarticular JIA encompasses two distinguished subtype: persistent oligoarthritis and extended oligoarthritis. To stop the inflammatory process with poor systemic side effects IAC therapy was proposed and now this is the first choice therapy.

## Aim

To evaluate the efficacy of the intra-articular corticosteroid therapy (IAC) in 62 patients affected by oligo-articular JIA.

## Patients and methods

62 patients affected by oligoarticular JIA that referred to our Departments in the period between December 2004 and June 2008, were included in the study. 34 cases (54%) had persistent oligoarthritis and the only affected joints were knee (45%) and ankle (9%). The remaining patients presented extended oligoarthritis and the involved joints, in addition to knee and ankle, were elbow (3%), wrist (3%), interphalangeal (6%). Triamcinolone hexacetonide and Methylprednisolone acetate were used. Patients were followed up for three years. MTX was started only if NSAIDs and IAC failed. Clinical pattern and serologic data, such as ESR and ANAs, were evaluated in order to correlate them with treatment response to predict the outcome of IAC procedure.

## Results

The early treatment is more likely to determine a protracted response. Remission time is longer in persistent oligoarthritis, while is limited in the extended form. In presence of ANA positivity, prolonged remission is less likely.

## Conclusions

IAC determines long-lasting remission in the persistent JIA while it induces symptomatic relieve in the extended form. It seems that the associated systemic treatment could prolong the benefit duration. If an extended articular involvement occurs early it is proper to start systemic therapy.
